# Comparison of response evaluation criteria in solid tumors and tumor regression grade in evaluating the effect of preoperative systemic therapy of gastric cancer

**DOI:** 10.1186/s12885-022-10125-1

**Published:** 2022-10-01

**Authors:** Ming-Yu Lai, Shi-Yang Kang, Yu-Ting Sun, Ting-Ting Quan, Shi-Xun Lu, Cai-Yun He, Zhi-Wei Zhou, Li-Qiong Yang, Hui-Yan Luo, Feng-Hua Wang, Yu-Hong Li, Rui-Hua Xu, Wen-Long Guan, Miao-Zhen Qiu

**Affiliations:** 1 Department of Medical Oncology, Sun Yat-Sen University Cancer Center; State Key Laboratory of Oncology in South China; Collaborative Innovation Center for Cancer Medicine, 651 Dongfeng Road East, Guangzhou, 510060 China; 2 Department of Anesthesiology, Sun Yat-Sen University Cancer Center; State Key Laboratory of Oncology in South China; Collaborative Innovation Center for Cancer Medicine, 651 Dongfeng Road East, Guangzhou, 510060 China; 3 Department of Image, Sun Yat-Sen University Cancer Center; State Key Laboratory of Oncology in South China; Collaborative Innovation Center for Cancer Medicine, 651 Dongfeng Road East, Guangzhou, 510060 China; 4 Department of Pathology, Sun Yat-Sen University Cancer Center; State Key Laboratory of Oncology in South China; Collaborative Innovation Center for Cancer Medicine, 651 Dongfeng Road East, Guangzhou, 510060 China; 5 Department of Molecular Diagnostics, Sun Yat-Sen University Cancer Center; State Key Laboratory of Oncology in South China; Collaborative Innovation Center for Cancer Medicine, 651 Dongfeng Road East, Guangzhou, 510060 China; 6 Department of Gastric Surgery, Sun Yat-Sen University Cancer Center; State Key Laboratory of Oncology in South China; Collaborative Innovation Center for Cancer Medicine, 651 Dongfeng Road East, Guangzhou, 510060 China; 7 Department of Experiment, Sun Yat-Sen University Cancer Center; State Key Laboratory of Oncology in South China; Collaborative Innovation Center for Cancer Medicine, 651 Dongfeng Road East, Guangzhou, 510060 China

**Keywords:** Gastric cancer, Preoperative therapy, Response evaluation criteria in solid tumors, Tumor regression grade, Immunotherapy

## Abstract

**Background:**

Both Response Evaluation Criteria in Solid Tumors (RECIST) and tumor regression grade (TRG) play key roles in evaluating tumor response. We analyzed the consistency of TRG and RECIST 1.1 for gastric cancer (GC) patients and compared their prognostic values.

**Methods:**

Patients with GC who received preoperative chemotherapy or chemoimmunotherapy and had records of TRG from December 2013 to October 2021 were enrolled retrospectively. TRG 0–1 and 2–3 are considered as corresponding to complete response (CR)/partial response (PR) and stable disease (SD)/progress disease (PD) in RECIST 1.1, respectively. The primary endpoints were disease-free survival (DFS) and overall survival (OS). The consistency of RECIST and TRG was examined by kappa statistics. Survival analysis was performed using the Kaplan Meier method.

**Result:**

One hundred fifty seven GC patients were enrolled, including 125 with preoperative chemotherapy and 32 with chemoimmunotherapy. Among them, 56 patients had measurable lesions. Only 19.6% (11/56) of the patients had consistent results between RECIST 1.1 and TRG. TRG was correlated with both OS and DFS (*P* = 0.02 and 0.03, respectively) while response according to RECIST1.1 was not (*P* = 0.86 and 0.23, respectively). The median DFS had not reached in the TRG 0–1 group and was 16.13 months in TRG 2–3 group. TRG 2–3 was associated with young age and peritoneal or liver metastasis. Besides, preoperative chemoimmunotherapy had a significantly higher pCR rate than chemotherapy alone (34.4% vs 8.0%, *P* < 0.001).

**Conclusion:**

TRG was in poor agreement with RECIST 1.1. TRG was better than RECIST 1.1 in predicting DFS and OS for GC patients who received preoperative therapy.

**Supplementary Information:**

The online version contains supplementary material available at 10.1186/s12885-022-10125-1.

## Introduction

Perioperative chemotherapy (PC) with radical surgery represents the gold standard of treatment for resectable locally advanced gastric cancer (GC). GC causes a large number of health and economic burdens worldwide and it is the second most common diagnosed cancer and leading cause of cancer-related death in China [1]. About 60% of the patients in China are diagnosed as locally advanced or metastatic diseases. Radical surgery is still the most promising therapy for resectable GC. To increase the rate of radical resection and lower the odds of distant metastasis or local recurrence, preoperative chemotherapy, including neoadjuvant chemotherapy and conversion chemotherapy, is widely used. Some studies have proved that preoperative therapy could improve long-term survival [2–4]. Both the Magic Trial and FNCLCC/FFCD study showed a survival benefit of perioperative chemotherapy, compared with surgery alone [2, 5]. More recently, these results were supported by the multicentric RESOLVE trial, which found that perioperative chemotherapy had a significantly better disease-free survival than adjuvant chemotherapy [6]. Even for stage IV patients, some of them may receive radical surgery after conversion chemotherapy. So, it is important to evaluate the effectiveness of preoperative chemotherapy. The most widely used criteria for evaluating tumor response to certain therapy in clinical practice is the Response Evaluation Criteria in Solid Tumors (RECIST) [7, 8]. Besides, to evaluate local tumor response histologically after chemotherapy, another approach called tumor regression grade (TRG) is often used. Though there are several TRG systems and no international standard, several studies have indicated the correlation between TRG and prognosis [9–12]. However, in clinical practice, we found that the results of TRG and RECIST were not always consistent. We wonder which method performs better in predicting the prognosis. Therefore, we designed the present study to verify the consistency of TRG and RECIST criteria and compare the prognostic value of TRG and RECIST 1.1 to preoperative therapy in GC patients.

## Methods

### Patient selection

In this study, the data of gastric cancer patients who received preoperative therapy between December 2013 and October 2021 at Sun Yat-sen University Cancer Center were retrospectively collected. The inclusion criteria were: 1. had received chemotherapy or chemotherapy combined with immunotherapy before surgery; 2. underwent gastrectomy surgery. The exclusion criteria were as follows: 1. underwent preoperative radiotherapy; 2. History of another malignancy within the last 5 years except cured basal cell carcinoma of skin and cured carcinoma in situ of the uterine cervix. Our study was performed in accordance with the Declaration of Helsinki protocols and was approved by the local Ethics Committee of SYSUCC and waived patient-specific consent.

### Evaluation system for tumor regression grade and RECIST

The overall survival (OS) was calculated from the date of diagnosis to death or the final follow-up date. The disease-free survival (DFS) was calculated from gastrectomy surgery to death or the final follow-up date or the date of recurrence/metastasis. The failure events of the survival analysis were death for OS and tumor recurrence or death for DFS. The censoring data was defined as not having observed a failure event and not knowing the exact survival time.

This study applied the National Comprehensive Cancer Network (NCCN) classification system to assess the tumor regression grade (TRG). TRG was defined by the percentage of viable cancer cells in the resected primary tumor: TRG 0 = complete response, no viable tumor cells including lymph nodes; TRG 1 = near complete response, single cancer cells or rare small groups of cells; TRG 2 = partial response, residual tumor cells with obvious tumor regression but the amount of cancer cells is more than that in TRG 1; TRG 3 = poor or no response, extensive residual cancer with no evident tumor regression [13, 14]. According to previous studies, pathological response was defined as TRG = 0 or 1 [15].

RECIST version 1.1 were applied in the present study: CR (complete response) means disappearance of all target lesions, PR (partial response) means the sum of the diameters of all targets lesions deceases ≥30%, PD (progressive disease) means the sum of the diameters of all targets lesions increases ≥20%, SD (stable disease) means insufficient shrinkage to qualify for PR or insufficient increase to qualify for PD) [7]. Patients who got CR or PR were categorized as clinical response (effective group) [16].

### Statistical analysis

SPSS ver. 22.0 software (IBM Corp., Armonk, NY) was used to perform statistical analysis in this study. Consistency of TRG and RECIST were analyzed using kappa statistics (kappa ≦ 0.40, poor agreement; 0.40 < kappa ≦ 0.60, moderate agreement; 0.60 < kappa ≦ 0.80, good agreement; and kappa > 0.80, excellent agreement) [17]. OS and DFS was estimated using the Kaplan-Meier method. The OS and DFS curves for different TRG groups and different RECIST groups were compared using the log-rank test. Chi-Square test or Fisher’s exact test were used as appropriate to compare the different neoadjuvant therapies as well as clinicopathological characteristics associated with different TRG groups and different RECIST groups. *P* values less than 0.05 were considered significant statistically.

## Results

### Clinicopathological characteristics

We finally collected a total of 157 patients with preoperative treatment and gastrectomy in the present study. 125 (79.6%) patients were administered preoperative chemotherapy, and 32 (20.4%) patients received preoperative chemotherapy combined with immunotherapy. Most patients were male (65.6%), and their median age was 64 years old (ranging from 24 to 78). Most primary tumors (72.0%) were gastric cancers, and 28.0% were in the esophageal-gastric junction. Five patients (3.2%) were mismatch repair deficient (dMMR), and 10 (6.4%) were human epidermal growth factor receptor-2 (HER-2) positive. Fifty patients (31.8%) were classified as diffuse type and 60 (38.2%) were classified as intestinal type.

The TRG scores were as follows: TRG 0 (*n* = 24, 15.3%); TRG 1 (*n* = 29, 18.65%); TRG 2 (*n* = 60 38.2%); TRG 3 (*n* = 44, 28.0%). Only 56 patients had measurable lesions, including PD (*n* = 1, 1.8%), SD (*n* = 25, 44.6%) and PR (*n* = 30, 53.6%). The patient characteristics are listed in Supplementary Table 1.

In all 157 patients, 44 (28.0%) showed progression, including local recurrence and distant metastasis. According to RECIST 1.1, 7 of them achieved PR after preoperative therapy and 13 got SD. The rest could not be evaluated since they had no targeted lesion. Of the 44 patients with progression after surgery, no one got pCR after preoperative therapy, 7 got TRG 1, 19 got TRG 2, and 18 got TRG 3.

Patients who got SD or PD after preoperative chemotherapy tended to have peritoneal metastasis (*P* < 0.05, Table 1). TRG 2 or 3 was associated with younger age, advanced clinical stage at diagnosis, and liver or peritoneal metastasis (All *P* < 0.05, Table 2).

**Table 1:** Clinicopathologic variables associated with RECIST 1.1

Characteristics		Effective	Ineffective	*P* value
		*N* = 30	*N* = 26	
**Age**	< 65	17 (48.6%)	18 (51.4%)	0.33
	> = 65	13 (61.9%)	8 (38.1%)	
**Gender**	Male	17 (56.7%)	13 (43.3%)	0.62
	Female	13 (50.0%)	13 (50.0%)	
**BMI**	< 18.5	1 (20.0%)	4 (80.0%)	0.08
	8.5–23.9	20 (66.7%)	10 (33.3%)	
	> 23.9	9 (42.9%)	12 (57.1%)	
**Position**	Gastric	20 (48.8%)	21 (51.2%)	0.24
	Esophageal–gastric junction	10 (66.7%)	5 (33.3%)	
**Lauren**	Diffuse	9 (40.9%)	13 (59.1%)	0.37
	Non-diffuse	15 (53.6%)	13 (46.4%)	
	NA	6 (100.0%)	0 (0%)	
**Liver metastasis**	Yes	2 (40.0%)	3 (60.0%)	0.87
	No	28 (54.9%)	23 (45.1%)	
**Lymph node metastasis**	Yes	7 (58.3%)	5 (41.7%)	0.76
	No	23 (52.3%)	21 (47.7%)	
**Peritoneal metastasis**	Yes	5 (29.4%)	12 (70.6%)	0.02
	No	25 (64.1%)	14 (35.9%)	
**MMR**	pMMR	24 (54.5%)	20 (45.5%)	0.47
	dMMR	0 (0%)	1 (100.0%)	
	NA	6 (54.5%)	5 (45.5%)	
**Differentiation**	Medium-low differentiation	19 (45.2%)	23 (54.8%)	0.16
	Medium-high differentiation	7 (77.8%)	2 (22.2%)	
	NA	4 (80.0%)	1 (20.0%)	
**HER-2**	Positive	3 (75.0%)	1 (25.0%)	0.66
	Negative	23 (50.0%)	23 (50.0%)	
	NA	4 (66.7%)	2 (33.3%)	
**Clinical N stage**	N1	3 (42.9%)	4 (57.1%)	0.86
	N2 N3 NA	13 (54.2%) 11 (45.8%) 3 (75.0%)	11 (45.8%) 9 (54.2%) 2 (25.0%)	
**Preoperative staging**	IIb	1 (50.0%)	1 (50.0%)	0.44
	IIIa	4 (50.0%)	4 (50.0%)	
	IIIb	6 (42.9%)	8 (57.1%)	
	IIIc	6 (85.7%)	1 (14.3%)	
	IV	12 (50.0%)	12 (50.0%)	
	NA	1 (100.0%)	0 (0%)	
**Histology**	Adenocarcinoma	26 (53.1%)	23 (46.9%)	0.29
	Squamous carcinoma	1 (100.0%)	0 (0%)	
	Signet ring cell carcinoma	0 (0%)	2 (100.0%)	
	Others	3 (75.0%)	1 (25.0%)	
**EBER**	Negative	28 (59.6%)	19 (40.4%)	0.30
	Positive	1 (25.0%)	3 (75.0%)	
	NA	1 (20.0%)	4 (80.0%)	
**Duration of preoperative therapy**	1–2 months	5 (55.6%)	4 (44.4%)	0.78
	2–3 months	13 (54.2%)	11 (45.8%)	
	3-4 months	7 (63.6%)	4 (36.4%)	
	Over 4 months	5 (41.7%)	7 (58.3%)	

Statistically significant *P* values are given in bold (*P* < 0.05); Liver metastasis, LN metastasis, and peritoneal metastasis are clinical staging before starting chemotherapy

*dMMR* deficiency of mis-match repair; Effective in TRG: got 0 or 1 in TRG; Effective in TRG: got 0 or 1 in TRG; *MMR* Mis-match repair, *pCR* pathological complete response, *PD* Progressive disease, *pMMR* proficiency of mismatch repair; Poor differentiation included the low differentiation and low-median differentiation; *PR* Partial response, *RECIST* Response evaluation criteria in solid tumors, *SD* Stable disease, *TRG* Tumor regression grade

**Table 2:** Clinicopathologic variables associated with TRG

Characteristics		Effective *N* = 53	Ineffective *N* = 104	*P* value
**Age**	< 65	23 (26.4%)	64 (73.6%)	0.03
	> = 65	30 (42.9%)	40 (57.1%)	
**Gender**	Male	36 (35.0%)	67 (65.0%)	0.66
	Female	17 (31.5%)	37 (68.5%)	
**BMI**	< 18.5	2 (13.3%)	13 (86.7%)	0.11
	8.5–23.9	36 (40.0%)	54 (60.0%)	
	> 23.9	15 (30.6%)	34 (69.4%)	
	NA	0 (0%)	3 (100.0%)	
**Position**	Gastric	38 (33.6%)	75 (66.4%)	0.96
	Esophageal–gastric junction	15 (34.1%)	29 (65.9%)	
**Lauren**	Diffuse	17 (34.0%)	33 (66.0%)	0.09
	Non-diffuse	18 (20.9%)	68 (79.1%)	
	NA	18 (85.7%)	3 (14.3%)	
**Liver metastasis**	Yes	0 (0%)	10 (100.0%)	0.02
	No	53 (36.1%)	94 (63.9%)	
**Lymph node metastasis**	Yes	2 (11.8%)	15 (88.2%)	0.06
	No	51 (36.4%)	89 (63.6%)	
**Peritoneal metastasis**	Yes	5 (14.7%)	29 (85.3%)	0.01
	No	48 (39.0%)	75 (61.0%)	
**MMR**	pMMR	32 (27.1%)	86 (72.9%)	1.00
	dMMR	1 (20.0%)	4 (80.0%)	
	NA	20 (58.8%)	14 (41.2%)	
**Differentiation**	Poor differentiation	24 (22.4%)	83 (77.6%)	0.07
	Medium-high differentiation	13 (38.2%)	21 (61.8%)	
	NA	16 (100.0%)	0 (0%)	
**HER-2**	Positive	3 (30.0%)	7 (70.0%)	1.00
	Negative	34 (27.4%)	90 (72.6%)	
	NA	16 (69.6%)	7 (30.4%)	
**Clinical N stage**	0	2 (40.0%)	3 (60.0%)	0.18
	1	9 (33.3%)	18 (66.7%)	
	2 3	31 (40.3%) 10 (21.7%)	46 (59.7%) 36 (78.3%)	
	NA	1 (50.0%)	1 (50.0%)	
**Preoperative staging**	I-II	8 (44.4%)	10 (55.6%)	< 0.01
	IIIa	11 (37.9%)	18 (62.1%)	
	IIIb	23 (45.1%)	28 (54.9%)	
	IIIc	4 (33.3%)	8 (66.7%)	
	IV	5 (11.6%)	38 (88.4%)	
	NA	2 (50.0%)	2 (50.0%)	
**Histology**	Adenocarcinoma	45 (33.%)	91 (66.9%)	0.79
	Squamous carcinoma	0 (0%)	1 (100.0%)	
	Signet ring cell carcinoma	5 (35.7%)	9 (64.3%)	
	Others	3 (50.0%)	3 (50.0%)	
**EBER**	Negative	30 (25.4%)	88 (74.6%)	0.17
	Positive	5 (45.5%)	6 (54.5%)	
	NA	18 (64.3%)	10 (35.7%)	
**Cycles of preoperative therapy**	1–3	40 (40.0%)	60 (60.0%)	0.06
	4–6	11 (24.4%)	34 (75.6%)	
	7–10	1 (10.0%)	9 (90.0%)	

Statistically significant *P* values are given in bold (*P* < 0.05)

*dMMR* deficiency of mis-match repair; Effective in TRG: got 0 or 1 in TRG; Effective in TRG: got 0 or 1 in TRG; *MMR* Mis-match repair, *pCR* pathological complete response, *PD* Progressive disease, *pMMR* proficiency of mismatch repair; Poor differentiation included the low differentiation and low-median differentiation; *PR* Partial response, *RECIST* Response evaluation criteria in solid tumors, *SD* Stable disease, *TRG* Tumor regression grade

### Consistency analysis of TRG and RECIST

We considered TRG 0 or 1 as effective and TRG 2 or 3 as ineffective. Likewise, we regarded RECIST CR or PR as effective and SD or PD as ineffective. Thirty one patients got consistent results in TRG and RECIST 1.1 (11 were both effective and 20 were both ineffective, respectively). Ninteen patients got effective in RECIST 1.1 and ineffective in TRG, and another 6 patients got effective in TRG and ineffective in RECIST 1.1. The kappa value was 0.132, which showed poor consistency. Considering the patients were with different TNM stages, we classified them as neoadjuvant therapy group and conversion therapy group. There were 123 patients (78.3%) in neoadjuvant therapy group and 34 patients (21.7%) in conversion therapy group and we analyzed the agreement between TRG and RECIST separately. The kappa values were 0.089 and 0.048 in the preoperative chemotherapy group and conversion therapy group respectively, which showed poor consistency.

Considering that TRG scores focus on regression of primary lesions, which could not be considered as target lesions in the RECIST system, we retrospectively calculated the change of thickness of primary lesions after preoperative therapy and compared the changes with their TRG scores in 80 patients. When using 30% shrinkage of thickness as the cut-off point, the change of thickness on CT can predict the TRG score better (kappa value = 0.263, *P* = 0.02).

### Survival analysis of TRG and RECIST

**Figure 1:**
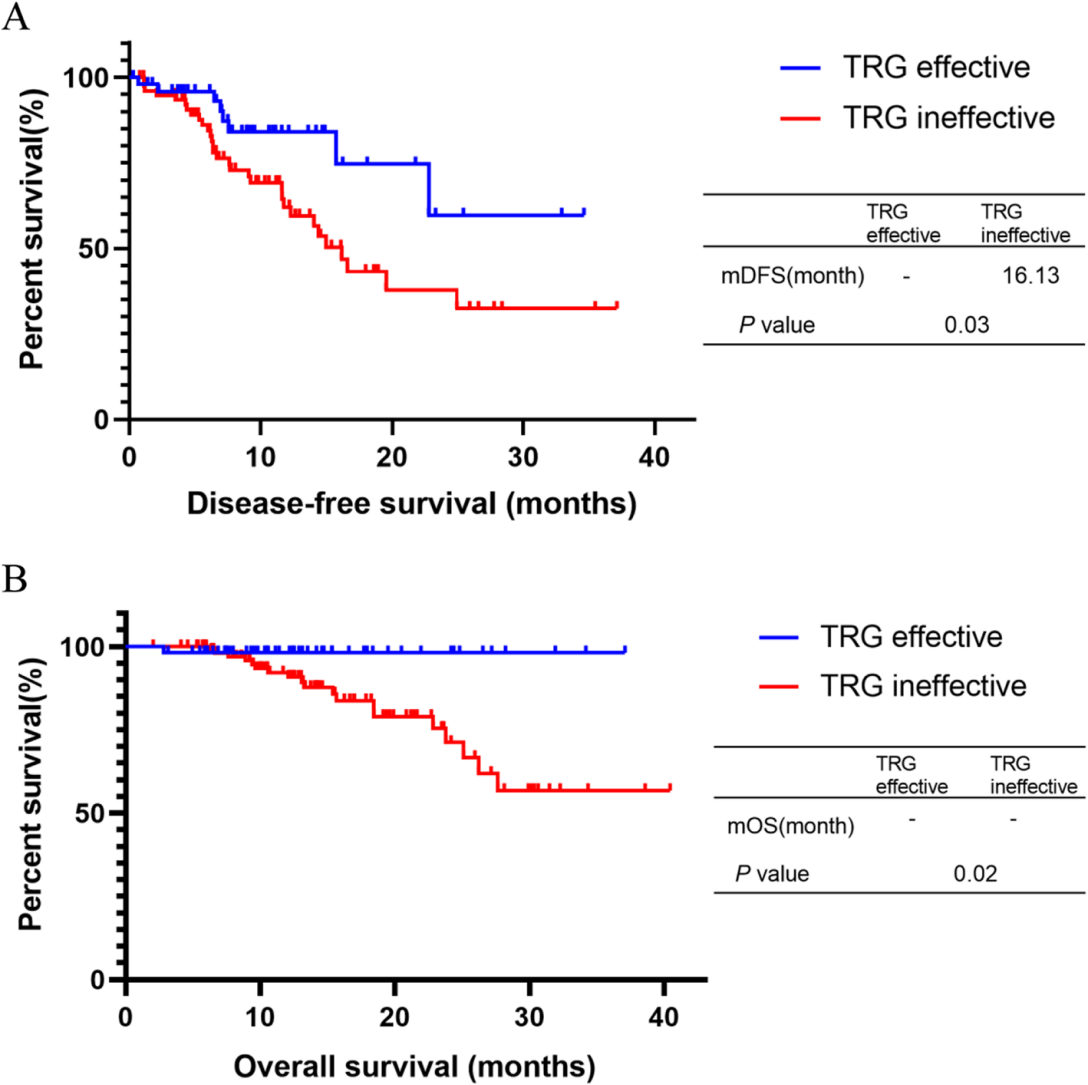
**Fig. 1 **Kaplan-Meier curves for different TRG groups. (**A**) Disease-free survival of TRG effective group and TRG  ineffective group, *P*=0.03. (**B**) Overall survival of TRG effective group and TRG ineffective group, *P*=0.02

Till Jan 28, 2022, 9 (17.0%) of 53 patients in the TRG effective group had disease progression, compared with 31 (29.8%) of 104 patients in the TRG ineffective group (*P* = 0.06). The median DFS was not reached vs. 16.13 months in TRG effective and ineffective group, respectively (*P* = 0.03). The median OS of both TRG 0–1 group and TRG 2–3 group were not reached (*P* = 0.02). The DFS and OS were significantly better in the TRG effective group (Fig. 1).

**Figure 2:**
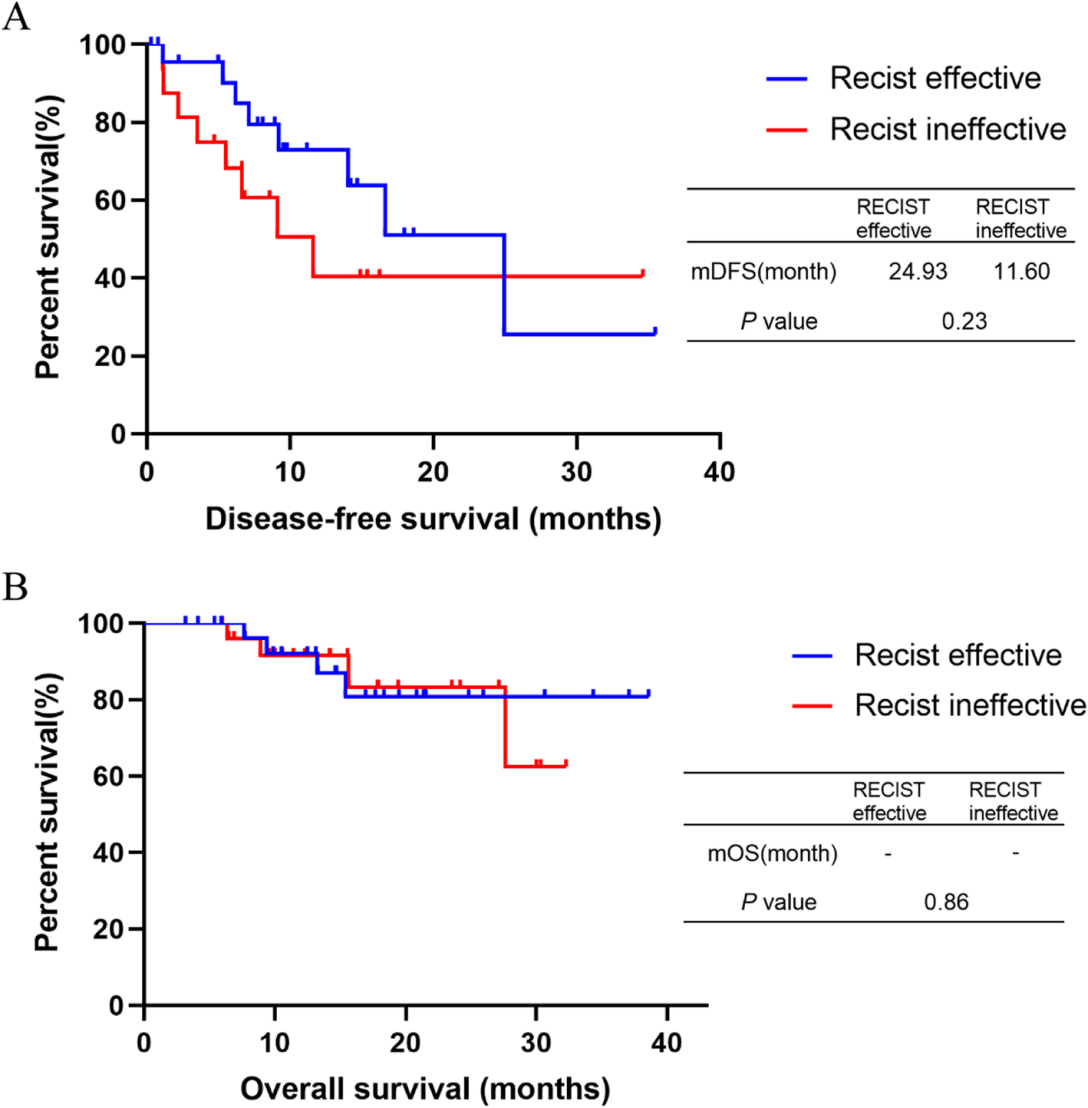
**Fig. 2 **Kaplan-Meier curves for different RECIST groups. (**A**) Disease-free survival of RECIST effective group and RECIST ineffective group, *P*=0.23; (**B**) Overall survival of RECIST effective group and RECIST ineffective group, *P*=0.86

Seven (23.3%) of 30 patients in the RECIST 1.1 effective group had disease progression, compared to eight (30.8%) of 26 patients in the ineffective group, *P* = 0.53. The median DFS for patients in the RECIST effective/ineffective group was 24.93 months and 11.60 months, respectively. The responses according to the RECIST assessment were not significantly correlated with either OS or DFS (*P* = 0.86 and 0.23, Fig. 2).

Furthermore, we divided patients with target lesions into 4 groups according to their responses in TRG and RECIST 1.1: both effective, only effective in TRG, only effective in RECIST 1.1, and both ineffective. Their OS and DFS were shown in Supplement Fig. 1. Patients who were only effective in TRG tended to have a better prognosis than those who were only effective in RECIST, though the difference was not significant (not reached vs. 24.93 months for DFS, respectively, *P* = 0.52;not reached vs. not reached for OS, *P* = 0.75) (Supplement Fig. 2).

Considering the preoperative treatment was sorted into neoadjuvant therapy and conversion therapy, we analyzed the survival according to TRG and RECIST respectively in each subgroup. It showed that the response of TRG tended to correlate with survival in both subgroups, though the *P* values were not significantly different (Supplement Fig. 3).

### Comparative analysis between different neoadjuvant therapies

Patients in the chemoimmunotherapy group had a higher pathological complete response (pCR) rate than those in the chemotherapy group (34.4% vs 8.0%, respectively, *p* = 0.001). The median DFS was 19.53 months and not reached for patients who received chemotherapy or chemoimmunotherapy, respectively. But the difference of DFS and OS between these two groups were not statistically significant (*P* = 0.91 for both OS and DFS) (Supplement Fig. 4). Patients who received chemoimmunotherapy also had a higher response rate (TRG 0 or 1) than those who only received chemotherapy (50.0% vs 29.6%, respectively, *P* = 0.03).

### Patients with pathologic complete response

A total of 21 patients (12.8%) got pCR (The follow-up time ranging from 3.13 months to 34.17 months). Among them, 5 (23.8%) got PR according to RECIST 1.1, and the rest had no target lesions. Five patients did not receive any treatment after surgery, and none of them suffered from recurrence or metastasis with. Seven patients (33.3%) had received chemotherapy combined with immunotherapy after surgery, and one of them had peritoneum metastasis 4 months after surgery. One patient (4.8%) had received immunotherapy alone. Eight patients (38.1%) had received postoperative chemotherapy (Supplement Table 2).

## Discussion

In the present study, we found that TRG and RECIST 1.1 got poor consistency. The possible reasons for this inconsistency may include: firstly, the evaluation targets of these two systems are different. TRG mainly evaluates primary lesions, while RECIST 1.1 focus on measurable target lesions (for example, lymph nodes, liver metastasis), which excluded primary gastric lesion. Hence, we tried to compare TRG with the degree of shrinkage of primary lesions on computed tomography (CT), which showed better consistency with TRG. Secondly, unlike other malignancies, gastric cancer is a tumor with high heterogeneity, which may lead to different responses to therapy between the primary site and lymph nodes or distant metastatic lesions. Thirdly, due to fibrosis, necrosis, or edema after preoperative therapy, the size of lesions may not change on imaging [18]. Finally, different imaging devices and measurement errors might also contribute to the inconsistency.

Furthermore, we found that TRG was a better prognostic factor than RECIST 1.1 for GC patients who received preoperative therapy. The TRG score for 84.1% of patients who developed disease progression was 2 or 3. Some previous studies demonstrated that TRG was a reliable prognostic factor for OS and DFS [19, 20]. Although the TRG systems they used were different from ours, their findings persuasively supported the conclusions of our present study. For patients who had inconsistent results between TRG and RECIST 1.1, we found that patients who were only effective in TRG tended to have better OS than those who only showed effectiveness in RECIST 1.1. Due to the small sample size, the difference was statistically insignificant. A larger sample size study was needed to confirm our findings.

Nowadays, perioperative chemotherapy has become the standard treatment for locally advanced GC patients, however, we found that only 35.7% (56/157) of the patients from our study had measurable lesions. About two-thirds of the patients could not be effectively evaluated by RECIST 1.1. Given the low consistency between RECIST 1.1 and TRG, the poor prognostic value and the limited application of RECIST1.1, novel parameters for imaging evaluation were warranted to better predict the response of preoperative therapy. A previous study found that Tumor Volume Reduction Rate (TVRR) was significantly associated with TRG in locally advanced rectal cancer patients [21]. CT perfusion parameter value was also found to improve the accuracy of response evaluation in esophageal cancer patients treated with neoadjuvant chemoradiotherapy [18]. Were there any feasible solutions to reduce those differences between radiology and pathology? Some attempts were made in the present study. We tried to take primary tumor into account and regarded patients whose gastric tumor had shrunken over 30% together as effective. The degree of consistency with TRG had risen, though not very strong. It was possible that the edema or fibrosis of the gastric wall after the preoperative treatment affected the measurement of thickness change. A similar comparison of the esophageal wall thickness after the preoperative treatment was reported in other available studies [18, 22, 23]. To sum up, after putting primary tumors into consideration, the sensitivity and accuracy of efficacy prediction could be higher than only using RECIST evaluation.

The rate of pCR was 12.8% in our study, but none of them was considered as CR by RECIST 1.1 before operation. A study demonstrated that FDG-PET/CT may be more accurate for the prediction of pCR [24]. pCR rate was significantly higher in patients who received chemoimmunotherapy than those with only chemotherapy. Chemoimmunotherapy can increase the ORR by 10–20% compared with chemotherapy alone in the first line setting for metastatic GC patients [25–27]. The pCR rate in neoadjuvant immunotherapy was higher than that in neoadjuvant chemotherapy in gastric cancer [28–30]. Another phase-2-study in the similar field called VESTIGE concluded that the combination of proper doses of immunotherapy can bring promising response rates in GC patients [31]. The value of chemoimmunotherapy in preoperative therapy still needs to be verified in prospective, randomized, controlled clinical trials.

Till now, there is no consensus on postoperative treatment for pCR patients. A recent cohort study revealed that gastric patients who were sensitive to preoperative chemotherapy tended to have a better prognosis if they would receive postoperative chemotherapy [32]. In our present study, we found a majority of pCR patients (76.2%) received postoperative chemotherapy or chemoimmunotherapy. Only one patient who received postoperative chemoimmunotherapy had distant metastasis 4 months after the operation. Further studies are needed to verify the role of postoperative therapy for pCR patients.

There were some inherent limitations in our study. Firstly, the sample size was small and only 56 patients had measurable lesions. Secondly, the regimens for preoperative treatment were different, including chemotherapy and chemoimmunotherapy. Finally, selection bias existed due to the specialty of the retrospective study. Despite the limitations above, we innovatively analyzed the postoperative treatment of pCR patients. Last but not least, we revealed the relationship between TRG and RECIST 1.1 directly, which was rare in previous studies.

In conclusion, our results suggested that pathological assessment (TRG) was in poor agreement with imaging assessment (RECIST 1.1), and TRG is more useful in predicting the effect of preoperative therapy and prognosis. Patients who achieved TRG 0 or 1 after surgery tended to have better OS and DFS compared to those who got TRG 2 or 3. We demonstrated that the addition of immunotherapy may increase the pCR rate than using chemotherapy alone. In addition, patients with pCR should also highly value postoperative therapy. Further studies with a larger sample size are needed to confirm our findings.

## Supplementary Information

**Additional file 1.**

**Additional file 2.**

**Additional file 3.**

**Additional file 4.**

**Additional file 5.**

**Additional file 6.**

## Data Availability

The datasets used and/or analyzed during the current study are available from the corresponding author on reasonable request.

## Abbreviations

RECIST: Response Evaluation Criteria in Solid Tumors
TRG: Tumor regression grade
GC: Gastric cancer
DFS: Disease free survival
OS: Overall survival
ORR: Overall response rate
PC: Perioperative chemotherapy
NCCN: National Comprehensive Cancer Network
CR: Complete response
PR: Partial response
PD: Progressive disease
SD: Stable disease
dMMR: Deficiency of mismatch repair
HER-2: Human epidermal growth factor receptor-2
pCR: Pathological complete response
CT: Computed tomography
TVRR: Tumor volume reduction rate
