# Supplementary material for: Comparison of response evaluation criteria in solid tumors and tumor regression grade in evaluating the effect of preoperative systemic therapy of gastric cancer
Source: BMC Cancer. 2022 Oct 1;22:1031. doi: 10.1186/s12885-022-10125-1 (PMC9526302; doi:10.1186/s12885-022-10125-1)
Supplement: Supplementary file 1 — Additional file 1. [file 12885_2022_10125_MOESM1_ESM.docx]

Supplement Table 1. Baseline patient characteristics

| Characteristics | Total(n=157) | （%） |
| --- | --- | --- |
| **Age**(years), median(range)  **Gender**  Male  Female  **BMI**  <18.5  18.5-23.9  >23.9  NA  **Preoperative treatment**  Chemotherapy  Chemotherapy combined with immunotherapy  **Position**  Gastric  Esophageal–gastric junction  **Lauren**  Diffuse  Intestinal  Mixed  NA  **MMR**  pMMR  dMMR  NA  **Differentiation**  Poor differentiated  Medium-low differentiation  Moderately differentiated  Medium-high differentiation  High differentiation  NA  **HER-2**  Positive  Negative  NA  [**Recurrence**](link:recurrence)  Yes  No  **pCR**  Yes  No  **RECIST**  PR  SD  PD  Non-CR/Non-PD  NA  **TRG**  0  1  2  3  **Death**  Yes  No | 64 （24-78）  103  54  15  90  49  3  125  32  113  44  50  60  26  21  118  5  34  74  33  34  0  0  16  10  124  23  44  113  21  136  30  25  1  63  38  24  29  60  44  20  137 | 65.6  34.4  9.6  57.3  31.2  1.9  79.6  20.4  72.0  28.0  31.8  38.2  16.6  13.4  75.2  3.2  21.7  47.1  21.0  21.7  0  0  10.2  6.4  79.0  14.6  28.0  72.0  13.4  86.6  19.1  15.9  0.6  40.1  24.2  15.3  18.5  38.2  28.0  12.7  87.3 |

dMMR: deficiency of mis-match repair; MMR: mis-match repair; pCR: pathological complete response; PD: progressive disease; pMMR: proficiency of mismatch repair; PR: partial response; RECIST: response evaluation criteria in solid tumors; SD: stable disease; TRG: tumor regression grade. Non-CR/Non-PD: means persistence of one or more non-target lesion(s) and/or maintenance of tumor marker lever above the normal limits.
