# Supplementary material for: Comparison of response evaluation criteria in solid tumors and tumor regression grade in evaluating the effect of preoperative systemic therapy of gastric cancer
Source: BMC Cancer. 2022 Oct 1;22:1031. doi: 10.1186/s12885-022-10125-1 (PMC9526302; doi:10.1186/s12885-022-10125-1)
Supplement: Supplementary file 2 — Additional file 2. [file 12885_2022_10125_MOESM2_ESM.docx]

Supplement Table 2. Clinical variables of pCR patients.

| Characteristics | Total (n=21) | (%) |
| --- | --- | --- |
| **Age**(years), median(range)  **Gender**  Male  Female  **BMI**  <18.5  18.5-23.9  >23.9  **Position**  Gastric  Esophageal–gastric junction  **Lauren**  Diffuse  Intestinal  Mixed  NA  **MMR**  pMMR  dMMR  NA  **HER-2**  Positive  Negative  NA | 65 (50-74)  16  5  1  16  4  13  8  4  6  4  7  8  0  13  0  11  10 | 76.2  23.8  4.8  76.2  19.0  61.9  38.1  19.0  28.6  19.0  33.3  38.1  0  61.9  0  52.4  47.6 |
| **RECIST**  PR  SD  PD | 5  0  0 | 23.8  0  0 |
| Non-CR/Non-PD | 5 | 23.8 |
| Not available  **Preoperative treatment**  Chemotherapy  Chemotherapy combined with immunotherapy | 11  10  11 | 52.4  47.6  52.4 |
| **Postoperative treatment** |  |  |
| None | 5 | 23.8 |
| Chemotherapy | 8 | 38.1 |
| Chemotherapy combined with Immunotherapy | 7 | 33.3 |
| Immunotherapy | 1 | 4.8 |
| **Recurrence or Metastasis** |  |  |
| Yes | 0 | 0 |
| No | 21 | 100 |

dMMR: deficiency of mis-match repair; MMR: mis-match repair; PD: progressive disease; pMMR: proficiency of mismatch repair; PR: partial response; RECIST: response evaluation criteria in solid tumors; SD: stable disease. Non-CR/Non-PD: means persistence of one or more non-target lesion(s) and/or maintenance of tumor marker lever above the normal limits.
