# Supplementary material for: Comparison of response evaluation criteria in solid tumors and tumor regression grade in evaluating the effect of preoperative systemic therapy of gastric cancer
Source: BMC Cancer. 2022 Oct 1;22:1031. doi: 10.1186/s12885-022-10125-1 (PMC9526302; doi:10.1186/s12885-022-10125-1)
Supplement: Supplementary file 3 — Additional file 3. [file 12885_2022_10125_MOESM3_ESM.pdf]

A

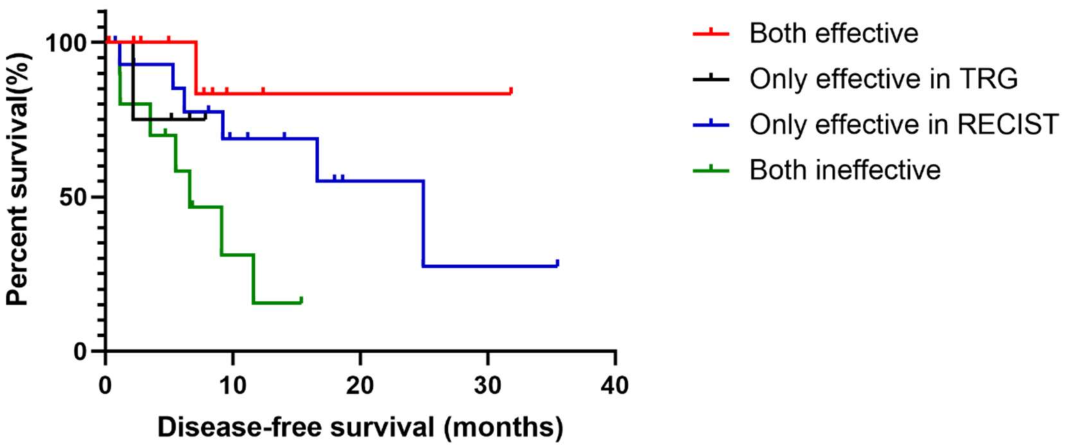

B

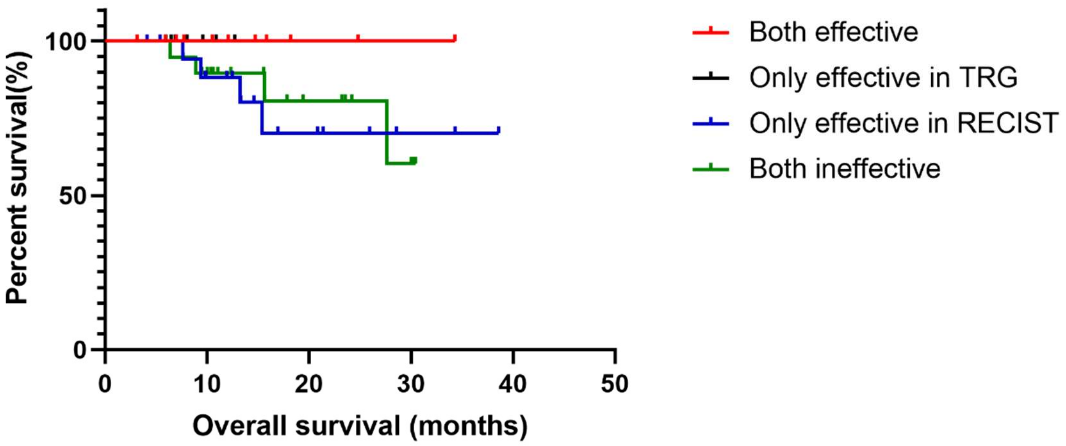

Supplement Figure 1. Kaplan-Meier curves for patients with measurable lesions. (B) Disease-free survival; (A) Overall survival.
