# Supplementary material for: Comparison of response evaluation criteria in solid tumors and tumor regression grade in evaluating the effect of preoperative systemic therapy of gastric cancer
Source: BMC Cancer. 2022 Oct 1;22:1031. doi: 10.1186/s12885-022-10125-1 (PMC9526302; doi:10.1186/s12885-022-10125-1)
Supplement: Supplementary file 4 — Additional file 4. [file 12885_2022_10125_MOESM4_ESM.pdf]

A

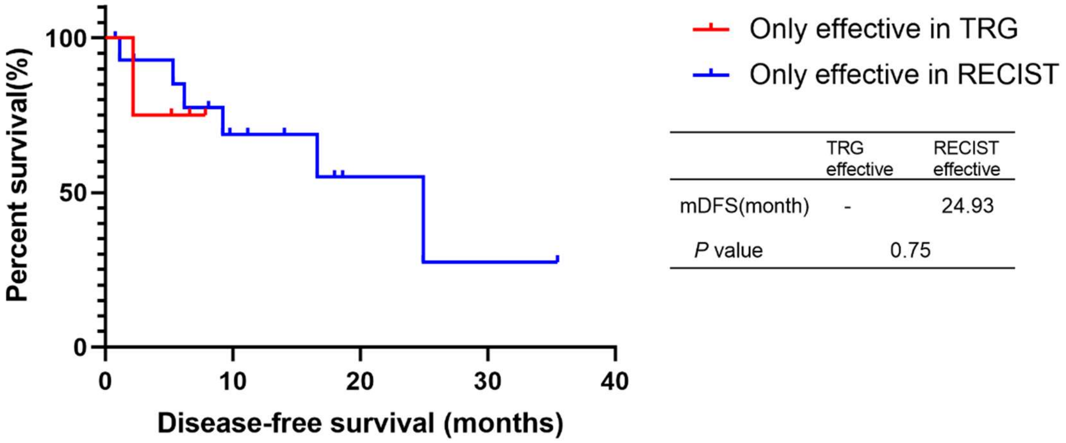

	TRG effective	RECIST effective
mDFS(month)	-	24.93
P value	0.75	

B

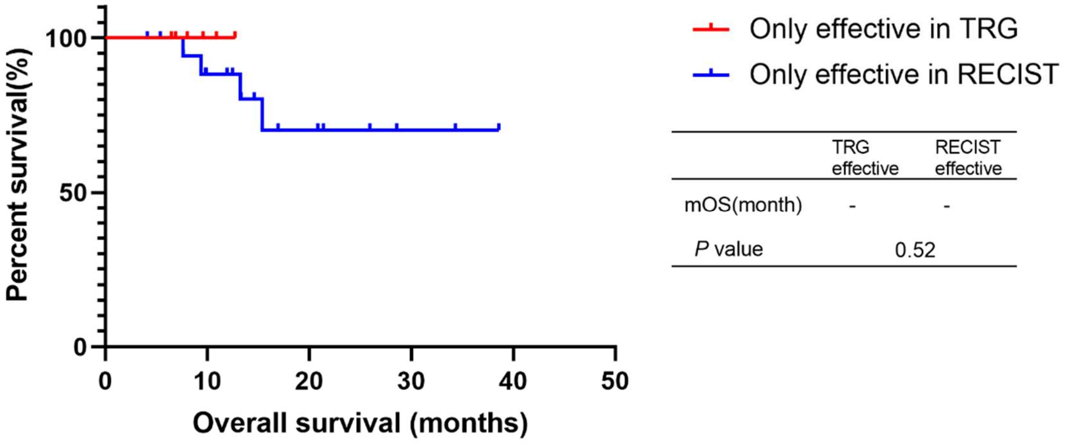

	TRG effective	RECIST effective
mOS(month)	-	-
P value	0.52	

Supplement Figure 2. Kaplan-Meier curves for patients with different responding conditions of TRG and RECIST. (A) Disease-free survival, *P*=0.75; (B) Overall survival, *P*=0.52.
