# Supplementary material for: Comparison of response evaluation criteria in solid tumors and tumor regression grade in evaluating the effect of preoperative systemic therapy of gastric cancer
Source: BMC Cancer. 2022 Oct 1;22:1031. doi: 10.1186/s12885-022-10125-1 (PMC9526302; doi:10.1186/s12885-022-10125-1)
Supplement: Supplementary file 6 — Additional file 6. [file 12885_2022_10125_MOESM6_ESM.pdf]

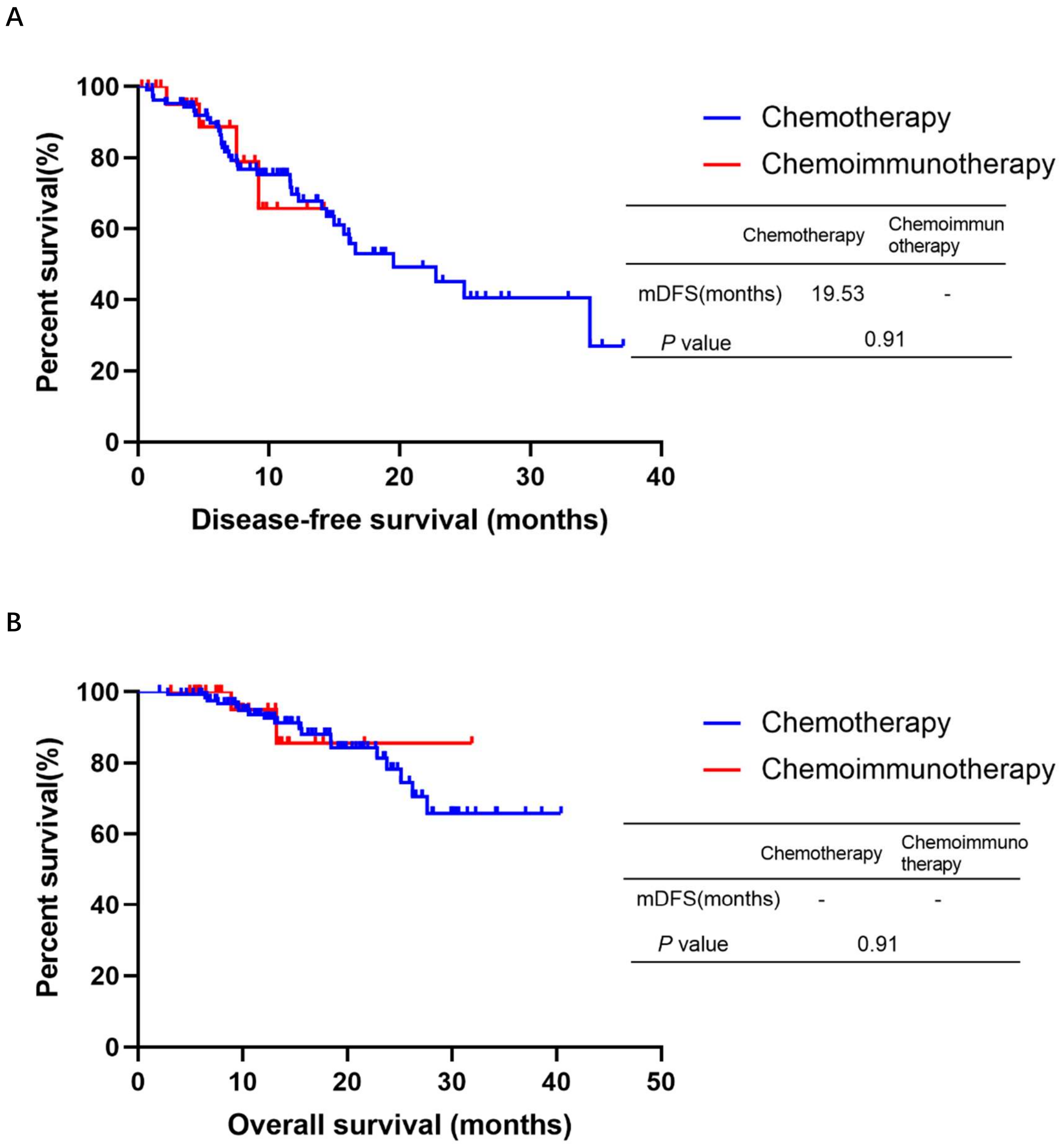

Supplement Figure 4. Kaplan-Meier curves for patients who received different preoperative treatments. (A) Disease-free survival, *P*=0.91; (B) Overall survival, *P*=0.91.
